# Spontaneous Osteonecrosis of Knee: A Case Report

**DOI:** 10.7759/cureus.44587

**Published:** 2023-09-02

**Authors:** Shivshankar Jadhav, Nareshkumar Dhaniwala, Ulhas Dudhekar, Mohit Dadlani, Abhiram A Awasthi

**Affiliations:** 1 Department of Orthopaedic Surgery, Datta Meghe Institute of Higher Education and Research, Wardha, IND

**Keywords:** subchondral bone, secondary arthritis, medial meniscus, osteochondral defect, osteonecrosis of knee

## Abstract

A 49-year-old man with no prior history of trauma, steroid use, or alcohol consumption presented with spontaneously developing progressive left knee discomfort that worsened after intense activity for 1.5 years. Normal x-rays indicated local discomfort along the joint line, and magnetic resonance imaging (MRI) revealed a T1-weighted hypointense line with bone infarcts in the medial and lateral condyle and the lower part of the left femur in addition to diffuse bone edema. Spontaneous osteonecrosis of the knee (SONK) was identified. Initially, he was treated conservatively with painkillers and calcium supplements. Then, the patient showed a significant improvement.

## Introduction

Ahlbäck et al. originally identified spontaneous osteonecrosis of the knee (SONK) in 1968 [1]. Initial symptoms of the illness include abrupt, atraumatic knee discomfort, with medial femoral condyle involvement often unilateral (about 90% of the time) [2]. Women are impacted three times more often than men, especially those who are 60 years or older [3]. Pain is frequent and can be quite debilitating, both during sleep and at rest. The most frequent physical exam finding is localized soreness to palpation across the afflicted region. Typically, patients will have mild synovitis, a tiny effusion, ligamentous stability, and little discomfort during range of motion. There seems to be a link between declining bone mineral density and the prevalence of SONK in women above 60 years and medial meniscus posterior root tears [4]. As many people who come with end-stage arthritis in the knees may have had SONK that went unrecognized, the real prevalence may be higher than currently stated [5].

On the basis of its etiology, it has recently been further divided into three subcategories: spontaneous (idiopathic, no known risk factor), postoperative (often after arthroscopy), and due to systemic illness (e.g., alcoholism, corticosteroid usage, etc.). There is no agreement on the cause of the disorder, unlike secondary osteonecrosis and post-arthroscopic osteonecrosis. Diagnosing and treating SONK due to its asymptomatic and insidious development might be difficult. Finally, it may result in secondary osteoarthritis, subchondral collapse, and the requirement for surgical treatment [6]. Thus, SONK can be managed conservatively with medications, and normal daily activities of living can be restored.

## Case presentation

**Figure 1:**
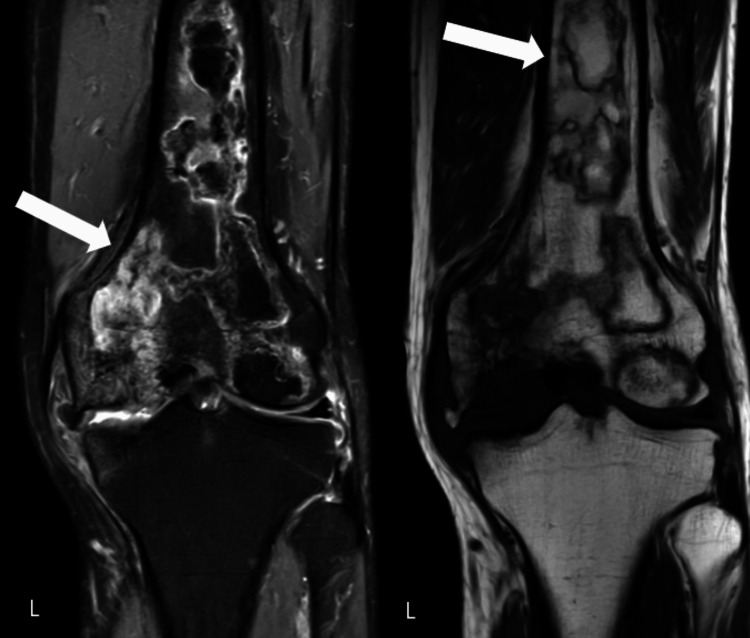
Magnetic resonance imaging of left knee This image shows bone edema and multiple bony infarcts in both condyles and the lower third of the left femur (marked with arrows).

A 49-year-old male patient who does not have a history of trauma or any other comorbidities has complained of abrupt onset for 1.5 years, escalating discomfort, and swelling over his left knee that worsens with activity. An examination of the body revealed joint line soreness. The patient gives no history of previous hospitalization or surgical intervention. Radiology was typical. Magnetic resonance imaging (MRI) revealed a T1-weighted hypointense line with bone infarcts in the medial and lateral condyle and the lower part of the left femur in addition to diffuse bone edema (Figure 1).

It was determined that the patient had SONK, also known as spontaneous osteonecrosis of the knee. Physical therapy in the form of exercises including hamstring and gastrocnemius-soleus stretches, hamstring and gluteus isometric exercises, active/passive knee full range of motion exercises, patella mobilization, gluteus, quadriceps, hamstring stretches, non-weight-bearing ambulation with crutches, diclofenac sodium (150 mg/day), and sodium alendronate (70 mg once a week) were used as the initial forms of conservative treatment for a period of 10 weeks. With medicine and physical therapy, the patient showed dramatic improvement. After six weeks, full weight-bearing activity was allowed. Around 10 weeks after the start of treatment, the patient was able to walk on his own.

## Discussion

Primary or spontaneous osteonecrosis of the knee (SONK), which typically affects one condyle in middle-aged or older females, and SONK, which is due to a known cause, are the two distinct conditions that frequently contribute to the osteonecrosis of the knee. The causes can be sickle-cell anemia or corticosteroid treatment, alcoholism, systemic disorders, radiation trauma, Caisson's disease, and chemotherapy. Secondary osteonecrosis of the knee more frequently affects younger patients and involves both condyles. Occasionally, these are silent lesions. In addition to this, increased knee discomfort and positive MRI results following arthroscopic surgery are signs of post-arthroscopy osteonecrosis [7,8].

The cause of SONK is yet unknown; it might be brought on by vascular damage and/or prior trauma [9]. According to the vascular theory, edema in a non-expandable compartment is caused by an unknown source of disturbance with the blood supply to the subchondral bone. As a result, the raised pressure in the bone marrow further reduces circulation, causes osseous ischemia, and lowers the marrow's signal strength on an MRI scan. Revascularization might lead to lesion healing and symptom relief if it happens before collapse. The theory that SONK is brought on by subchondral insufficiency fractures is supported by recent studies [10].

Localized soreness around the medial femoral condyle is the most frequent physical examination finding. When radiography is initially negative, especially when symptoms are transient, the diagnosis is frequently overlooked at this point. Over time, it develops a typical half-moon-shaped sclerosis with a radiolucent core close to the joint surface, followed by an imprint of the joint surface [11]. The pre-radiographic stage of SONK can be diagnosed using an MRI. Findings include fluid-filled cleft under the subchondral bone plate, flattening of the subchondral bone plate, hypointense signal in the subarticular marrow, and subchondral marrow edema. It is possible to monitor treatments using MRI.

Koshino's radiological classification of the lesions allows for the division of the condition into four stages (Table 1) [9]. Stages 1 and 2 are treated conservatively for up to six months until the extent of the lesion and its progression are determined.

**Table 1:** Koshino's radiological classification of the lesions

Stage	Description
Stage I	Radiographs show no anomalies
Stage II	Medial femoral condyle (MFC) flattening or radiolucent oval-shaped area in the subchondral region
Stage III	Growth of the radiolucent region surrounded by a sclerotic halo
Stage IV	Osteophytes and osteosclerosis seen around tibial and femoral condyles

Analgesics and bisphosphonates are used for treatment. Small lesions recover effectively, albeit slowly forming degenerative alterations may cause modest symptoms. Later stages that do not respond to conservative care necessitate surgical treatment. For the treatment of SONK, various progressive management methods have been developed, such as arthroscopic debridement, core decompression, osteochondral autograft transplantation (OAT), bone marrow stimulation with drilling or microfracture, high tibial osteotomy (HTO), unicompartmental knee arthroplasty, osteochondral allograft transplantation, and total knee arthroplasty. The development of SONK has also been linked to certain studies after arthroscopic surgery for a meniscal injury [12]. According to one idea, arthroscopic meniscectomy reduces the meniscus's ability to bear weight, which causes microtrauma and, in turn, may eventually result in SONK or SPONK, a process that is similar to that of osteoporosis [13].

To better understand the pathophysiology of the condition, Hussain et al. performed a comprehensive study in 2019 that reexamined the suggested causes of SONK. The researchers proposed that damage to the posterior medial meniscus root alters normal knee biomechanics by increasing tibiofemoral contact pressures, which in turn causes the subchondral insufficiency fractures observed in SONK [14]. Here, the researchers discovered a significant link between meniscal tears and unprovoked osteonecrosis of the knee. In the included investigations, it was discovered that meniscal tears occurred in 50%-100% of individuals with SONK, with the degree of medial meniscus extrusion according to the stage and volume of SONK lesions [15]. The authors also suggested a change in terminology, believing that subchondral insufficiency fractures of the knee (SIFK) would better depict the illness pathophysiology than the term "SONK" [14].

There is not much research that looks at the natural progression and long-term effects of SONK. Previous research has revealed that the size of the lesion does, in fact, affect the condition's prognosis, with larger lesions indicating a higher chance of osteoarthritis [16]. These findings were confirmed by Juréus et al. who studied SONK patients with a mean follow-up of 15 years. They concluded that when 40% or more of the joint surface was damaged, osteoarthritis was likely to occur. Non-operative treatments for SONK may be successful if discovered in its early stages [6]. Patients are prone to develop end-stage osteoarthritis if they are left undiagnosed. Due to the disease's deceptive onset and vague symptoms, spontaneous osteonecrosis of the knee can be difficult to diagnose. Care is required to rule out both intra-articular pathology and referred pain from concomitant hip disease [17]. Clinical conditions such as osteochondritis dissecans, shifting bone marrow edema, transient osteoporosis, secondary osteonecrosis or bone contusion, and concealed fractures can all be distinguished from SONK with the use of a thorough history and physical examination and advanced imaging techniques.

## Conclusions

Our male patient had a very unusual case of SONK that included the medial and lateral condyles as well as the shaft of the femur. The status of the patient can significantly improve with conservative care, which includes physical therapy and medicines such as bisphosphonates and non-steroidal anti-inflammatory drugs. If conservative care fails, several surgical procedures are also accessible if they are needed in the near future.
